# Unamplified and Real‐Time Label‐Free miRNA‐21 Detection Using Solution‐Gated Graphene Transistors in Prostate Cancer Diagnosis

**DOI:** 10.1002/advs.202205886

**Published:** 2022-12-08

**Authors:** Minghua Deng, Zhanpeng Ren, Huibin Zhang, Ziqin Li, Chenglong Xue, Jianying Wang, Dan Zhang, Huan Yang, Xianbao Wang, Jinhua Li

**Affiliations:** ^1^ Hubei Collaborative Innovation Center for Advanced Organic Chemical Materials Key Laboratory for the Green Preparation and Application of Functional Materials Ministry of Education Hubei Key Laboratory of Polymer Materials School of Materials Science and Engineering Hubei University Wuhan 430062 P. R. China; ^2^ School of Computer Science and Information Engineering Hubei University Wuhan 430062 P. R. China; ^3^ Department of Urology Tongji Hospital Tongji Medical College Huazhong University of Science and Technology Wuhan 430030 P. R. China

**Keywords:** label‐free miRNA‐21 detection, prostate cancer, real‐time detection, solution‐gated graphene transistors

## Abstract

The incidence of prostate cancer (PCa) in men globally increases as the standard of living improves. Blood serum biomarker prostate‐specific antigen (PSA) detection is the gold standard assay that do not meet the requirements of early detection. Herein, a solution‐gated graphene transistor (SGGT) biosensor for the ultrasensitive and rapid quantification detection of the early prostate cancer‐relevant biomarker, miRNA‐21 is reported. The designed single‐stranded DNA (ssDNA) probes immobilized on the Au gate can hybridize effectively with the miRNA‐21 molecules targets and induce the Dirac voltage shifts of SGGT transfer curves. The limit of detection (LOD) of the sensor can reach 10^−20^ M without amplification and any chemical or biological labeling. The detection linear range is from 10^−20^ to 10^−12^ M. The sensor can realize real‐time detection of the miRNA‐21 molecules in less than 5 min and can well distinguish one‐mismatched miRNA‐21 molecule. The blood serum samples from the patients without RNA extraction and amplification are measured. The results demonstrated that the biosensor can well distinguish the cancer patients from the control group and has higher sensitivity (100%) than PSA detection (58.3%). Contrastingly, it can be found that the PSA level is not directly related to PCa.

## Graphical abstract

An ultrasensitive and rapid quantification detection of the early prostate cancer‐relevant biomarker miRNA‐21 is designed based on gate‐functionalized SGGT. The LOD can reach 10^−20^ M and the linear range is from 10^−20^ to 10^−12^ M. The fastest response time is 3 min. The biosensor directly detects miRNA‐21 in clinical serum samples to discriminate prostate cancer patients.

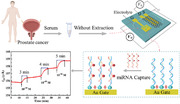

## 1 Introduction

PCa is a refractory cancer that increases with the age of men globally.^[^ ^1^ ^]^ Early detection and treatment of PCa may improve the survival rate to 99%, if the patients have localized prostate cancer.^[^ ^2^ ^]^ Common prostate cancer screening tests include PSA detection in serum, direct rectal examination (DRE), and image‐logical examination (MRI). PSA detection has been included in routine medical examinations in some countries. A PSA level above 4 ng mL^−1^ is regarded as a high risk of PCa. The PSA level range from 4 to 10 ng mL^−1^ (or 1 to 10 ng mL^−1^, different from age) is a so‐called gray zone owning to false positive and negative diagnoses, and the diagnostic sensitivity is just 58.3%.^[^ ^2^, ^3^ ^]^ These methods are imperfect for early diagnosis and bring discomfort to patients (especially elderly men). Thus, there is an obvious need for a novel diagnostic tool and biomarkers that are patient‐friendly and can offer tumor heterogeneity information with high accuracy. Among the diverse kinds of cancer biomarkers, miRNA is an OncomiR gene that can be used as a diagnostic tumor biomarker and in cancer evaluation at an early stage.^[^ ^4^, ^5^, ^6^, ^7^ ^]^ The unamplified and real‐time detections are challenges to the label‐free miRNA detection in early prostate diagnosis as miRNA strands are very short length (22 nucleobases) and low in concentration (approximately femtomolar level and lower) in body fluids. In addition to the conventional approaches of northern blotting analysis,^[^ ^8^ ^]^ quantitative real‐time polymerase chain reaction (qRT‐PCR),^[^ ^9^ ^]^ and microarray technology,^[^ ^10^ ^]^ surface plasmon resonance (SPR)^[^ ^11^ ^]^ and fluorescent platforms^[^ ^4^, ^12^, ^13^ ^]^ are also applied for the miRNA detection. These methods suffer from complex operation, long detection times, and require a specialized laboratory and specialized operators.

Electrochemical detection is a possible approach due to femtomolar level sensitivity and relatively low cost.^[^ ^14^, ^15^, ^16^, ^17^, ^18^, ^19^, ^20^ ^]^ Electrochemical transistors have been considered to fulfill the portable device requirements of high‐throughput, ultrasensitive, cost‐effective, rapidly, and real‐time detection without amplification, and any chemical or biological labeling.^[^ ^21^ ^]^ Diverse kinds of sensors, including DNA,^[^ ^22^, ^23^ ^]^ protein,^[^ ^24^, ^25^ ^]^ cell,^[^ ^26^ ^]^ neurotransmitter,^[^ ^27^, ^28^, ^29^ ^]^ virus,^[^ ^30^, ^31^ ^]^ glucose,^[^ ^32^, ^33^ ^]^ glycans,^[^ ^34^, ^35^ ^]^ ion,^[^ ^36^, ^37^, ^38^ ^]^ and growth factor sensors^[^ ^39^ ^]^ are designed based on transistors. SGGTs have been utilized in diverse biosensors due to their excellent biocompatibility, chemical stability, high mobility, and the ability to work at less than 1 V. SGGTs have been used in the detection of let‐7b,^[^ ^40^, ^41^ ^]^ miRNA‐4484,^[^ ^42^ ^]^ and other common kinds of miRNA.^[^ ^43^, ^44^ ^]^ Their detection sensitivity is normally from ≈10^−18^−10^−6^ M. These SGGT biosensors immobilized the bio‐probes directly on the conducting graphene channel surface and tested the Dirac voltage shift alone as the effective electrical signal. Pristine graphene is a hydrophobic material and has no functional group; crosslinker biomolecules were commonly used to attach probes.^[^ ^40^, ^41^, ^43^ ^]^ The single‐layer graphene channel is very sensitive; the nonspecific adsorption will cause graphene channel electrochemical doping and then induce transfer curve shift along the vertical current axis,^[^ ^40^, ^41^, ^42^, ^43^ ^]^ which will result in detection failure through the current measurement. MiRNA‐21 is a vastly studied oncomiR gene.^[^ ^45^ ^]^ It is also a prostate cancer‐relevant biomarker that is overexpressed in blood.^[^ ^4^ ^]^ Studies on miRNA‐21 detection in prostate cancer are predominantly electrochemical and optical detection.^[^ ^1^, ^17^, ^46^ ^]^ There are still several deficiencies in miRNA‐21 detection for early prostate cancer clinical diagnosis using SGGT‐based biosensors.

In this contribution, we constructed an unamplified and real‐time label‐free miRNA SGGT biosensor for early prostate cancer diagnosis. The ssDNA probe was designed and modified on the Au gate electrode surface to decrease nonspecific adsorption on the graphene channel. The ssDNA probes can hybridize effectively with the miRNA‐21 molecules targets and induce the Dirac voltage shifts of SGGT transfer curves. The sensors can realize the miRNA‐21 molecules detection, and the sensitivity of the sensor can reach 10^−20^ M without amplification and any chemical or biological labeling. The detection linear range is from 10^−20^ to 10^−12^ M. The sensor can realize the real‐time detection of miRNA‐21 molecules in less than 5 min at the low concentration of attomolar level and can well distinguish one‐mismatched miRNA‐21 molecule. The blood serum measurements demonstrated that the biosensor can well distinguish the cancer patients from the control group and has higher sensitivity than PSA detection.

## 2 Results and Discussion

### 2.1 Design and Characterization of SGGT‐Based miRNA Biosensor

**Figure 1:**
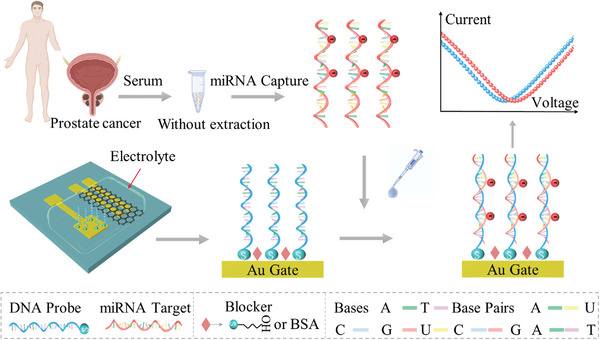
Schematic diagram of unamplified detection of label‐free miRNA‐21 using the ssDNA‐functionalized SGGTs biosensor.

**Figure** **1** depicts the schematic diagram of the unamplified detection of label‐free miRNA‐21 using ssDNA‐functionalized SGGTs biosensor. The chemical vapor deposition (CVD)‐grown graphene was transferred to the glass substrate with the source and drain Au electrodes and acted as the conductive channel. The graphene film quality was characterized by the Raman spectrum, as depicted in Figure S1, Supporting Information. It can be found that 2D and G peak intensity ratio was ≈1.93, indicating that the CVD‐grown graphene film had high quality. The mechanical stability and high mobility of the graphene can guarantee good electrical conductivity of the SGGTs biosensor.^[^ ^47^, ^48^ ^]^ To decrease the nonspecific adsorption on the graphene channel, the ssDNA probe is designed to be immobilized on the solid Au gate electrode for the biosensing interface.^[^ ^49^ ^]^ The probe ssDNA can capture the negative miRNA‐21 and induce Dirac point shifts which can be measured by the source meter. To determine if the probes are immobilized on the gate electrode surface, X‐ray photoelectron spectroscopy (XPS) was conducted, as depicted in Figure S2, Supporting Information. The N, P, and S element peaks at 400.6, 133.6, and 162 eV demonstrated successful ssDNA probe immobilization.^[^ ^50^ ^]^ The ssDNA probe modification was also characterized by cyclic voltammetry (CV), as depicted in Figure S3, Supporting Information. When the ssDNA was modified on the gate electrode, the oxidation/reduction peak became weak. This is because the ssDNA molecules had low redox activity compared to Au. It implied that ssDNA probe was immobilized on the gate electrode. To verify the hybridization of the ssDNA probe and the miRNA‐21 target, miRNA‐21 targets were labeled with fluorescence Cy3 molecules. Figure S4, Supporting Information, depicts the inverted fluorescence microscope images of the blank gate, the ssDNA‐modified gate, and the ssDNA‐modified gate electrode with the hybridization of miRNA‐21 targets. Red fluorescence could be observed on the gate electrode after the hybridization of the miRNA‐21 targets with the probes. However, no fluorescence was observed on the blank and ssDNA‐modified gate electrode surfaces. It indicates that ssDNA probe plays a key role in miRNA‐21 detection.

### 2.2 Sensing Mechanism of SGGT‐based miRNA Biosensor

**Figure 2:**
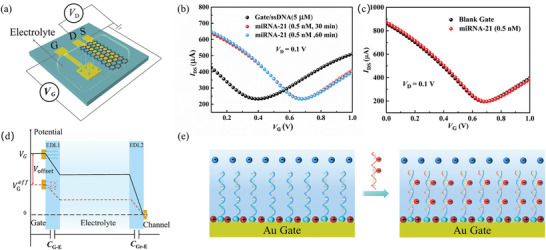
a) Configuration of the ssDNA‐functionalized SGGT‐based miRNA sensor b) Transfer curves of the device with probe immobilization for miRNA (0.5 nM) sequences incorporation. c) Transfer curves of the device without probe immobilization for miRNA sequence incorporation. d) The variety of the gate voltage across the EDLs. e) Schematic diagram of charge variation before and after miRNA‐21 solution incorporation.

To explore the biosensor sensing mechanism, the transfer curve of the sensor was tested by applying the voltage to the three electrodes as depicted in **Figure** **2**a. *V*_G_ was applied on the Au gate (G) electrode and *V*_D_ was applied on the Au Drain (D) electrode. The Au source electrode was grounded as the reference voltage. The RNase‐free electrolyte solution containing 1 mM Mg^2+^ (MgCl_2_ diluted in ×0.1 PBS buffer) immersed the gate and channel surface with a poly (dimethylsiloxane) (PDMS) cavity. The incorporation of Mg^2+^ can help the ssDNA probe maintain active conformation to promote the hybridization rate.^[^ ^51^, ^52^ ^]^ The thiol‐ssDNA probes shorter than 24 bases were anchored on the Au gate surface in dendritic configuration.^[^ ^53^, ^54^ ^]^ When the miRNA‐21 target solution was incorporated to the PDMS well, hybridization between ssDNA probe and miRNA target occurred and the Dirac point of the transfer curve shifted towards a more positive gate voltage, as depicted in Figure 2b. Compared to 30 min hybridization time, the Dirac point voltage of the transfer curve demonstrated an obvious shift toward the positive gate voltage. However, no Dirac point voltage shift was observed after one hour. It indicates that the hybridization time is less than 30 min. To verify that the sensor Dirac point voltage shift (Δ*V*_Dirac_) was induced by the hybridization of the ssDNA probe and miRNA‐21 molecule on the gate electrode, the control experiment was conducted without ssDNA probe modification. Figure 2c depicts the transfer curves of the device without ssDNA probes modification before/after incorporation of the miRNA‐21 solution to the test well. No obvious shift of Dirac point voltage was observed. It suggests that the Dirac point shifts originate from the capture of the miRNA target by the ssDNA probe. Figure 2d depicts the equivalent circuit model of two double electrical layers (EDLs) which can be formed when *V*_G_ is applied. *C*_G‐E_ is the capacitance of the gate/electrolyte interface (EDL1), and *C*_Gr‐E_ is the capacitance of the electrolyte/graphene interface (EDL2). Figure 2e depicts the schematic diagram of charge variation before and after the incorporation of miRNA‐21 solution. The miRNA‐21 molecule is electronegative due to the phosphate backbone. When the miRNA‐21 target molecules are captured by the ssDNA probes on the gate electrode surface and hybridized with the ssDNA probes, it is equivalent to applying a negative potential on the gate electrode, called offset voltage (*V_offset_*). The gate voltage *V*_G_ can control the change of the channel current *I*_DS_ at a fixed drain voltage *V*_D_ using the following equations.^[^ ^23^, ^25^, ^55^ ^]^

(1) $$\begin{array}{c} \begin{array}{c} I_{\mathrm{DS}}\approx \frac{W}{L}\mu C_{i}\left|V_{G}^{\mathrm{eff}}-V_{\mathrm{Dirac}}-\frac{V_{D}}{2}\right|\cdot V_{D}\left(\mathrm{when}\left|V_{D}\right|\leq \left|V_{G}^{\mathrm{eff}}-V_{\mathrm{Dirac}}\right|\right) \end{array} \end{array}$$

(2) $$V_{G}^{e\mathrm{ff}}=V_{G}+V_{offset}$$

(3) $$V_{offset}=\frac{\Delta Q}{C_{i}A}$$

where *W* and *L* are the channel width and length; *µ* is the graphene carrier (electron or hole) mobility; *C*_i_ is the total capacitance; *V*_Dirac_ is the charge neutrality point voltage; $V_{G}^{e\mathrm{ff}}$ is the effective gate voltage, Δ*Q* is the variable amount of gate electrode induced by miRNA‐21 target molecules, and A is the gate electrode area. According to Equation (2), when the miRNA‐21 target molecules are captured, the transfer curve of the device will shift toward the direction of the positive voltage axis because the *V*_offset_ is a negative value. Therefore, the miRNA sensor sensing mechanism is attributed to the potential variety on the gate electrode induced by the electronegativity of the miRNA.

### 2.3 Sensitivity of the SGGT‐Based miRNA Biosensor and Real‐Time Detection

**Figure 3:**
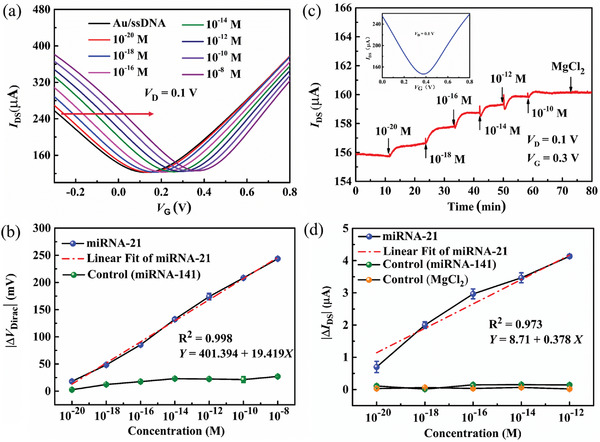
a) Transfer curves of the biosensor at the diverse miRNA‐21 concentrations. b) | Δ*V*_Dirac_| of the biosensor at the diverse miRNA‐21 and miRNA‐141 concentrations. c) Real‐time channel current response (*I*_DS_) of the biosensor at diverse miRNA‐21 concentrations. d) Δ*I*_DS_ of the biosensor at the diverse MgCl_2_, miRNA‐141, and miRNA‐21 concentrations.

We investigated the quantitative relation of Δ*V*_Dirac_ and the miRNA concentrations. **Figure** **3**a depicts the transfer curves of the sensor with the ssDNA probe modification at the diverse miRNA‐21 target concentrations. The transfer curves shifted towards the positive gate voltage direction with the increase in miRNA‐21 target concentrations. It is because miRNA carries a negative charge in the electrolyte solution. When the DNA probes hybridized with the miRNA targets, it induced potential change on the Au gate electrode. The Δ*V*_Dirac_ values extracted from the transfer curves were 11, 61, 111, 151, 180, 210, and 250 mV for the miRNA target concentration varying from 10^−20^ to 10^−8^ M. As depicted in Figure 3b, the Δ*V*_Dirac_ could be linearly fitted very well in the concentration range from 10^−20^ to 10^−8^ M, and the *R^2^* was 0.998, which indicates that the SSGTs have an excellent linear range in the plot of |Δ*V*_Dirac_| – Log *C*_miRNA‐21_. From Figure 3a,b, the LOD of the biosensor can reach 10^−20^ M. Generally, the fabricated devices with CVD‐grown graphene films were sealed into a container and stored at 4 °C. As depicted in Figure S5, Supporting Information, the Dirac point voltages were relatively stable for four weeks. The stability of the sensor with/without the ssDNA probes was tested in an electrolyte solution every two hours. Figures S6a,b depict the transfer curves of the biosensor with/without the ssDNA probes with time. No obvious Dirac point voltage shifts were observed in 6 h, which indicates that the sensor has good stability. To investigate the selectivity of the sensor, the electrical response of the biosensor to the interferent miRNA‐141(14‐base mismatched) was measured, as depicted in Figure S7, Supporting Information. The transfer curves of the biosensor demonstrated a very small shift when the concentration of miRNA‐141 increased from 10^−20^ to 10^−8^ M. The Δ*V*_Dirac_ values can be extracted from the transfer curves in Figures 3a and S7, Supporting Information. It is obvious that the response of the biosensor to miRNA‐21 is higher than those to miRNA‐141 when the concentration of miRNA‐21 exceeds 10^−17^ M. It indicates that the biosensor has the strong anti‐interference ability of miRNA‐141.

Figure 3c depicts the real‐time channel current responses of the biosensor to the incorporation of miRNA‐21 targets with concentrations from 10^−20^ to 10^−10^ M. When the miRNA‐21 targets were incorporated to the PDMS well, *I*_DS_ increased, and then reached steady values post completion of the hybridization procedures. It can be found that the device demonstrates an excellent current response when the concentration of miRNA‐21 targets reaches 10^−20^ M. The limit of detection for the biosensor can reach 10^−20^ M which is significantly lower than those reported previously, as depicted in Table S1, Supporting Information. To further, determine the detection time of the sensor, the time scale was magnified, as depicted in Figure S8, Supporting Information. The detection times for the concentrations of 10^−20^, 10^−18^, and 10^−16^ M were ≈3, 4, and 5 min, respectively. With the concentration increase of miRNA‐21 targets, the detection time also increased. The |Δ*I*_Dirac_| versus the logarithm of the miRNA‐21 concentration (*C*_miRNA‐21_) can be plotted and linearly fitted, as depicted in Figure 3d. |Δ*I*_Dirac_| and *C*_miRNA‐21_ depicted a good linear relationship in the concentration range of 10^−20^−10^−12^ M, and the *R* ^2^ was 0.973. The current responses of the biosensor to MgCl_2_ and miRNA‐141 interferences were measured, as depicted in Figure 3d. It can be found that the current responses of the biosensor to miRNA‐21 targets were apparently higher than those of MgCl_2_ and miRNA‐141 interferences. It implies that the current response signals from the miRNA‐21 targets are very reliable. These results indicate that the SGGT‐based miRNA‐21 biosensor has very low LOD and can realize real‐time detection.

### 2.4 Selectivity of the SGGT‐Based miRNA Biosensor

**Figure 4:**
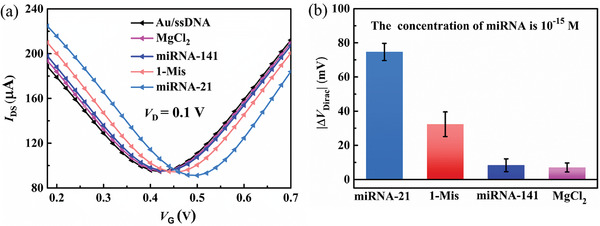
Selectivity of the sensor a) Transfer curves of the biosensor post incorporation of MgCl_2_, interferent (miRNA‐141:14‐base mismatched, 1‐Mis: one base mismatched), and target miRNA‐21. b) Dirac point voltage shifts extracted from the transfer curves.

For investigating the selectivity of the biosensor, early prostate cancer biomarker miRNA‐21, and other noncomplementary miRNAs (including 14‐base mismatched: miRNA‐141 and one‐base mismatched: 1‐Mis) were detected. MgCl_2_ was used as the blank control. As depicted in **Figure** **4**a, there was an obvious miRNA‐21 Dirac voltage shift, while the same concentration of other miRNAs and MgCl_2_ induced a few Dirac point voltage shifts. In these interferents, 1‐Mis generated the largest Dirac point voltage shift. However, the Dirac point voltage shift for the complementary miRNA‐21 target was four times higher than that for the 1‐Mis targets, as depicted in Figure 4b. The results demonstrated that the proposed biosensor has excellent specificity.

### 2.5 Unamplified miRNA‐21 Testing of Clinical Serum Samples

**Figure 5:**
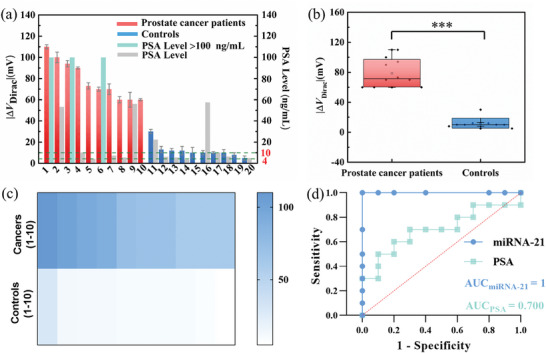
Detection of miRNA‐21 in the clinical serum samples a) Dirac point voltage shifts and PSA levels of diverse clinical serum samples. b) A significant difference of Dirac point voltage shifts among prostate cancer patients and controls. c) Heat map depicting the degree of Dirac point voltage shifts across 20 clinical serum samples. d) ROC curves of SGGT‐based miRNA biosensor response to miRNA‐21 in clinical serum samples and PSA diagnosis in the hospital.

The designed SGGT‐based miRNA biosensor was also applied for detection in clinical samples. The information of all clinical samples is enlisted in Table S2, Supporting Information. Before detection, all clinical samples were diluted 1000‐fold. Unlike the previous work of applying preheating at 95 °C for 15 min to release the entrapped miRNAs from membrane vesicles,^[^ ^56^ ^]^ the clinical samples were directly^[^ ^41^ ^]^ tested without nucleic acid extraction and PCR amplification. Although the clinical serum samples contain other background biomolecules, miRNA‐21 from prostate cancer patients could be directly detected by measuring the Dirac point voltage shift of the SGGT miRNA biosensor. **Figure** **5**a,b depict that the Dirac point voltage shifts (output electrical signal response) of the clinical samples from prostate cancer individuals were higher than those of the control group with a statistically significant difference (*p* < 0.001). This indicates that the designed biosensor can distinguish the patients from the control group. In the control group, only one benign prostatic hyperplasia (BPH) patient (sample 11#) with a PSA level of 22.312 ng mL^−1^ induced a relatively larger Dirac point voltage shift of 30 mV which was just a half shift of the lowest value (60 mV) of the PCa patient. Therefore, this method can satisfactorily screen out the PCa patient through an SGGT‐based miRNA biosensor. Although it is essential to check more clinical samples, from Figure 5b,c,d, it can still be found that the diagnostic accuracy rate of the biosensor can reach 100% which is significantly higher than the PSA tests (58.3%). The receiver operating characteristic curve (ROC) was applied, and the area under ROC of SGGT‐ based biosensor response to miRNA‐21 was 1, implying an excellent diagnostic accuracy rate. However, the area under ROC of the PSA level was 0.700 which indicated low sensitivity. Contrastingly, it could also be found that the PSA level is not directly related to PCa. From Table S2, Supporting Information, it can be observed that the PCa patients were elder men aged between 50 and 85 years. This group has higher requirements for diagnostic accuracy and friendliness. Therefore, this biosensor demonstrates great potential for early PCa diagnosis.

## 3 Conclusion

In summary, we have successfully fabricated an unamplified, real‐time, ultra‐sensitive, and cost‐effective miRNA biosensor based on SGGT through thiol ssDNA probe modification. The LOD of the sensor can reach 10^−20^ M and the linear range is from 10^−20^ to 10^−12^ M. The detection time is ≈5 min when the target concentration is not higher than 10^−16^ M. The sensor also demonstrates excellent selectivity. It can well distinguish the one‐base mismatched miRNA. The sensing mechanism of the biosensor is attributed to the transfer curve shift induced by the hybridization of the ssDNA probe on the gate electrode and the miRNA targets. SGGT sensor allows the direct detection of the clinical serum samples without nucleic acid extraction, labeling, and PCR amplification. It can effectively distinguish cancer patients from non‐cancer patients. Because the SGGT sensors are easy to be integrated and adopt the electrical signal measurement, they can be developed as portable detection equipment. The SGGT sensors demonstrate great potential in the early detection of cancer.

## 4 Experimental Section

### Materials

The synthetic ssDNA probe with high‐performance liquid chromatography (HPLC)‐purified was purchased from GenScript Biotech Co. Ltd. (Nanjing, China). The probe sequences with sulfhydryl at the 5’ end, Cy3‐labeled miRNA‐21, and one‐base mismatched miRNA were designed and acquired from GenScript Biotech Co., Ltd (Nanjing, China). The miRNA‐21 (target) and noncomplementary miRNA‐141^[^ ^57^ ^]^ were also acquired from GenScript Biotech Co., Ltd. The ssDNA and miRNA solution were stored at −20 °C. The base pairs of all nucleotide sequences are enlisted in Table S3, Supporting Information. Tris (2‐carboxyethyl) phosphine hydrochloride (TCEP: 10 mM) and 10 × Phosphate Buffered Saline (PBS, pH = 7.4, enzyme free) were obtained from Gibco (Shanghai, China). The DEPC water (DNase, Rnase free) was purchased from Beyotime Biotech Co.Ltd. (Shanghai, China). RNaseZap decontamination solution was acquired from Thermo Fisher Co.Ltd. (Shanghai, China). Acetone, ethanol, and ferric chloride were obtained from the Sinopharm company (Shanghai, China). Magnesium chloride hexahydrate (MgCl_2_), 6‐mercapto‐1‐hexanol (MCH), Poly (methyl methacrylate) (PMMA), and Poly (dimethylsiloxane) (PDMS) were purchased from Aladdin Co. Ltd. (Shanghai, China). Glass substrates were purchased from Guluo company (Luoyang, China). The CVD‐grown single‐layer graphene was acquired from SixCarbon Tech. company (Shenzhen, China). The clinical samples of prostate cancer patients and healthy volunteers were provided by the Tongji Hospital (Wuhan, China), which was approved by the ethics committee of the hospital. The experiments were carried out with the full, informed consent of the subjects.

### Fabrication of a SGGT Biosensor

The fabrication process of the SGGT device is the same as our previous work.^[^ ^58^ ^]^ Au electrodes (Cr, 3 nm; Au,30 nm) were deposited on a glass substrate. The side length of the square gate electrode was 3 mm. The channel length (*L*) and width (*W*) were 0.2 and 6.0 mm, respectively. The CVD‐grown graphene film (length, 6 mm; width, 2 mm) was transferred onto the channel using the wet chemical method. To get a clean Au solid surface, the electrodes were washed ultrasonically twice before transferring graphene film.

### Surface Modification of the Au Gate Electrode

To avoid the influence of impurities on the modification, the gold electrodes were treated with piranha solution (H_2_SO_4:_ H_2_O_2_, v: v = 7: 3) for 1 min, followed by washing with ethanol and deionized water. All devices were treated with RNaseZap decontamination solution. The Au gate electrodes were then immersed in Thiol‐ssDNA solution (1 µM, 10 µL) for 12 h in an electro‐heating standing‐temperature cultivator at 28 °C and subsequently in 0.1 mM MCH solution for 1 h to block the unbound acting sits on the surface. During detection of the clinical samples, the unbound acting sites were blocked with BSA (1%) to reduce nonspecific adsorption.

### Detection of miRNA using SGGT Biosensor

The SGGT biosensors were immersed in several solutions (complementary miRNA‐21, 14‐bases mismatched miRNA‐141, and one‐base mismatched miRNA‐21) for 30 min. A blank control test was conducted with MgCl_2_ diluted in ×0.1 PBS buffer. The incorporated miRNA target solution was serially diluted from high concentration to low concentration. Then, 100 µL buffer solution were incorporated into the PDMS well as an electrolyte, The target solution was incorporated from low to high concentration with 1 µL. The transfer curves were measured before and after the miRNA‐DNA interaction by sweeping the *V*_G_ from 0 to +1 V with a 0.1 V *V*_D_. The Dirac voltage (Δ *V*_Dirac_) shifts induced by the analytes were used as a sensing signal. Concurrently, the channel response currents were measured to conduct real‐time detection. The change channel current (Δ*I*) of the SGGT biosensor induced by the analytes was used as the real‐time sensing signal.

### Collection of Clinical Samples for miRNA‐21 Testing

Clinical samples were collected from Tongji Hospital (Wuhan, China), as depicted in Table S2, Supporting Information. Ten patients were diagnosed with prostate cancer. Five patients were diagnosed BPH. Two were diagnosed with prostatitis and three were healthy individuals. The upper serum was collected from the blood by centrifuging at 3000 RPM for 10 minutes and stored at −80 °C. The frozen serum samples were defrosted at −20 °C, 4 °C, and 28 °C in turn. Then the clinical samples were diluted on a biological ultra‐clean table before testing. This research was approved by Tongji Hospital Ethics Committee (approval number: 2020HGRY016).

## Conflict of Interest

The authors declare no conflict of interest.

## Supporting information

Supporting Information

## Data Availability

Research data are not shared.
