# Supplementary material for: Unamplified and Real‐Time Label‐Free miRNA‐21 Detection Using Solution‐Gated Graphene Transistors in Prostate Cancer Diagnosis
Source: Adv Sci (Weinh). 2022 Dec 8;10(4):2205886. doi: 10.1002/advs.202205886 (PMC9896035; doi:10.1002/advs.202205886)
Supplement: Supplementary file 1 — Supporting Information [file ADVS-10-2205886-s001.pdf]

Supporting Information

Unamplified and real-time label-free miRNA-21 detection using solution-gated graphene transistors in prostate cancer diagnosis

Minghua Deng [a], Zhanpeng Ren [a], Huibin Zhang [a], Ziqin Li [a], Chenglong Xue [a], Jianying wang [a], Dan Zhang [b, *], Huan Yang [c, *], Xianbao Wang [a] and Jinhua Li [a, *]

[a] *Hubei Collaborative Innovation Center for Advanced Organic Chemical Materials, Key Laboratory for the Green Preparation and Application of Functional Materials, Ministry of Education, Hubei Key Laboratory of Polymer Materials, School of Materials Science and Engineering, Hubei University, Wuhan 430062, P. R. China.*

[b] *School of Computer Science and Information Engineering, Hubei University, Wuhan 430062, P. R. China.*

[c] *Department of Urology, Tongji Hospital, Tongji Medical College, Huazhong University of Science and Technology, Wuhan 430030, P. R. China.*

* Corresponding authors

Email: jinhua_li@hubu.edu.cn (J. Li), Leaf769@163.com (D. Zhang) and yhpz123@163.com, (H. Yang)

Table of Contents

Raman spectrum of the CVD-grown graphene on Si substrate (Figure S1) ………… S3

XPS characterization of the Au gate electrode (Figure S2) …………………………S3

CV characterization of the Au gate electrode (Figure S3) ……………………………S4

Inverted fluorescence microscope of the Au gate electrode (Figure S4) ……………S4

Storage stability of the fabricated devices (Figure S5) … ……………………………S5

Stability testing of the biosensor in electrolyte solution (Figure S6) …………………S5

Transfer curve of the target miRNA-141 (Figure S7) ……………… ………………S6

Magnified time scale of channel current response of miRNA-21 concentrations from 10 zM to 100 aM (Figure S8) ……………… ……………………………………………S6

Comparison of different miRNA detection methods (Table S1) …………… … …… S7

Comparison of medical diagnosis and miRNA-21 detection results from 20 individuals (Table S2) ………………………………………………………………………… … S8

The ssDNA and miRNA sequences used in this work (Table S3) ………………… …S8

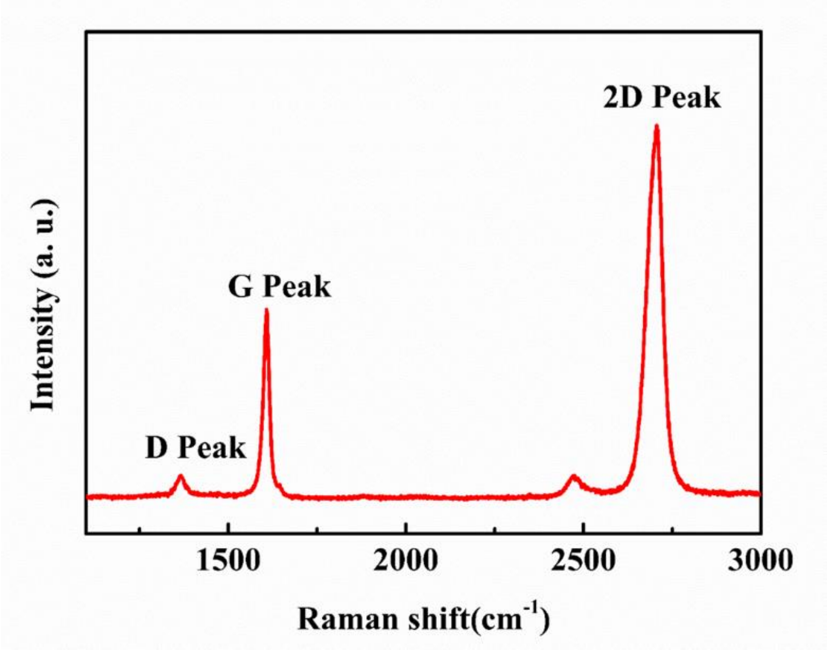

Figure S1 Raman spectrum of the CVD-grown graphene on Si substrate.

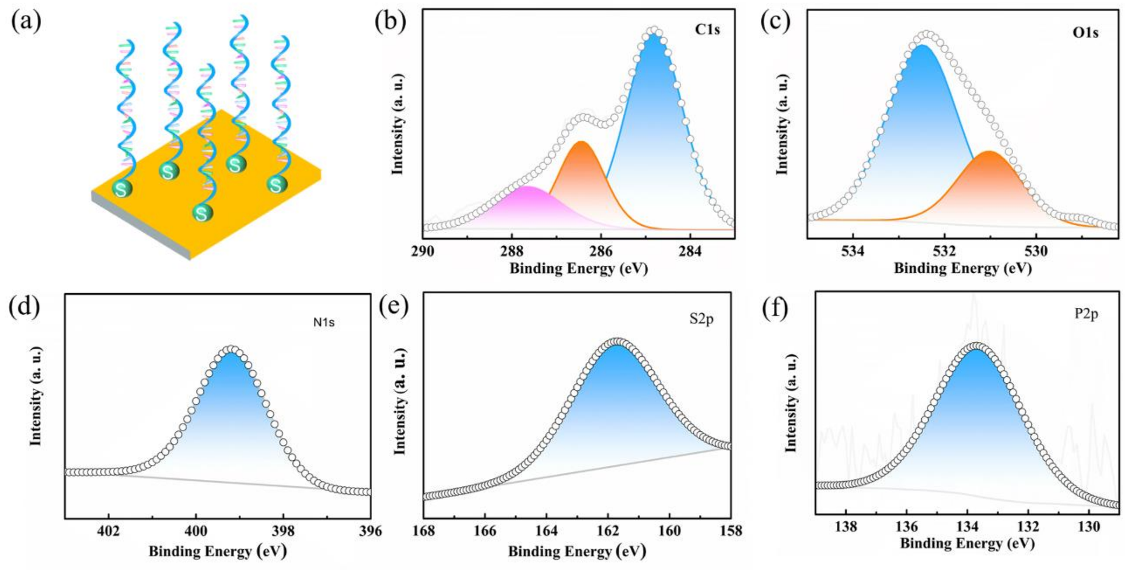

Figure S2 Functionalization and XPS characterization of the Au gate electrode. (a) Au gate with the immobilization of DNA probe. (b-f) characterization of Au gate with the immobilization of DNA probe. X-ray photoelectron spectroscopic spectra of C, O, N, S, P elements, respectively.

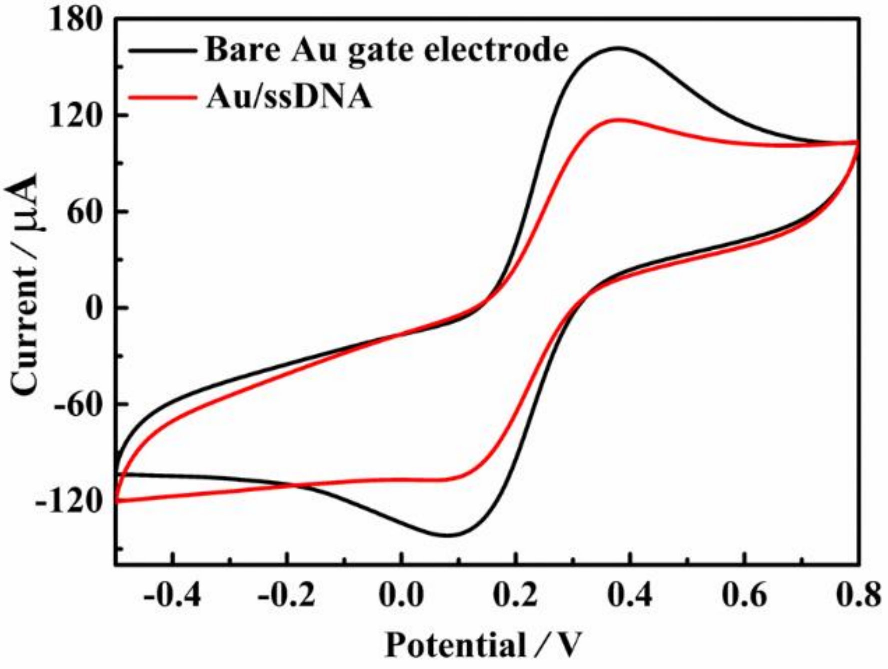

Figure S3 CV characterization of bare Au gate electrode, and the modification of ssDNA probe.

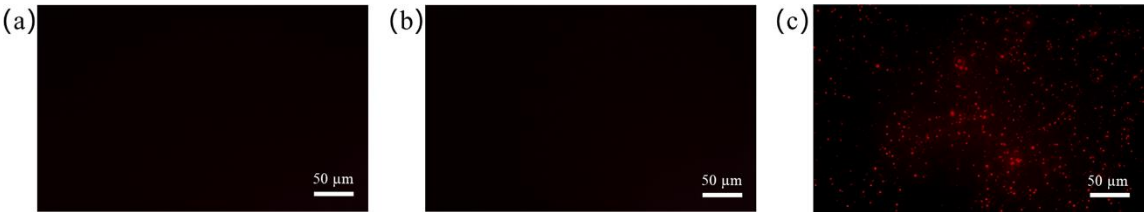

Figure S4 (a) Inverted fluorescence microscope of the blank Au gate electrode. (b) Inverted fluorescence microscope of the Au gate electrode After the probe DNA immobilization. (c) Inverted fluorescence microscope of the Au gate electrode after the hybridization of thiol-ssDNA probe and miRNA target.

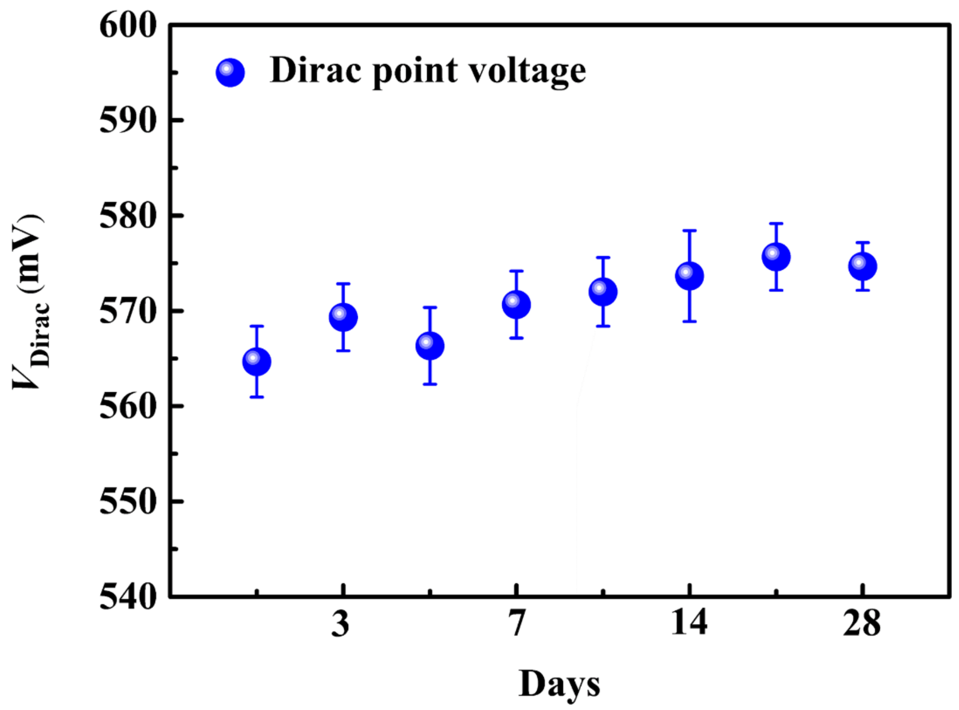

Figure S5 Storage stability of the fabricated devices with CVD-grown graphene films.

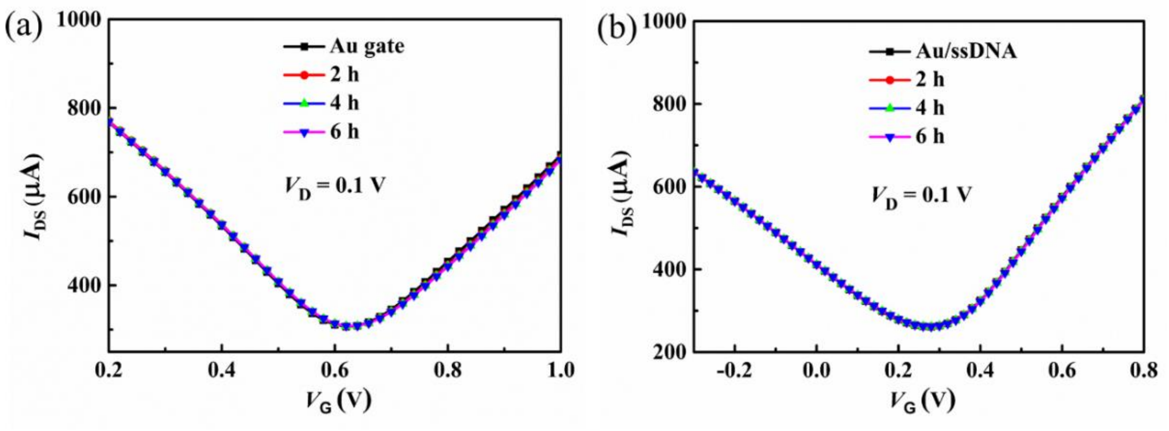

Figure S6 Stability testing of the biosensor in electrolyte solution. (a) SGGT-based biosensor without the gate modification. (b) SGGT-based biosensor with the gate modification.

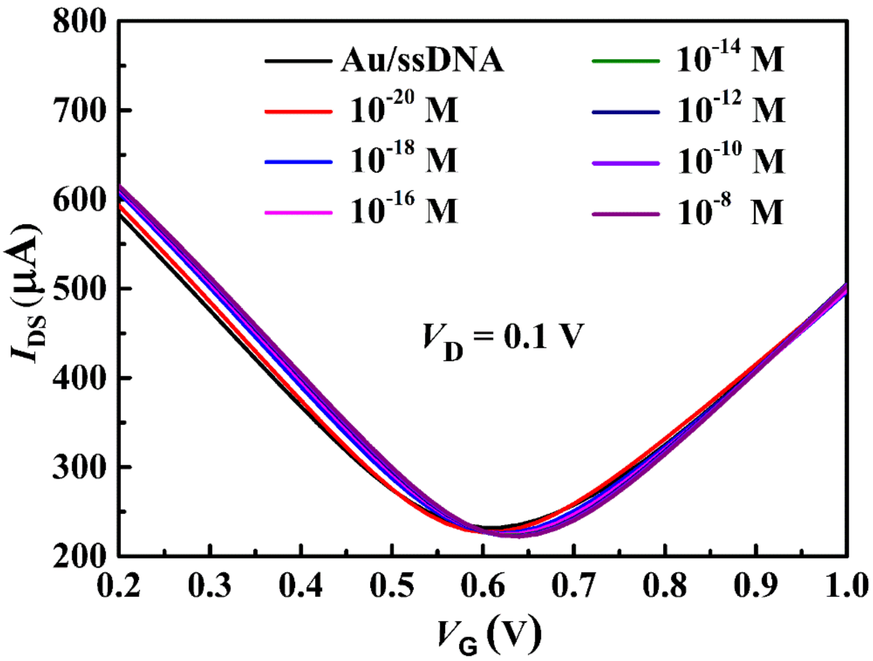

Figure S7 Transfer curve of the sensor with the ssDNA probe immobilization after the addition of the different concentrations of 14-mismatched miRNA (miRNA-141).

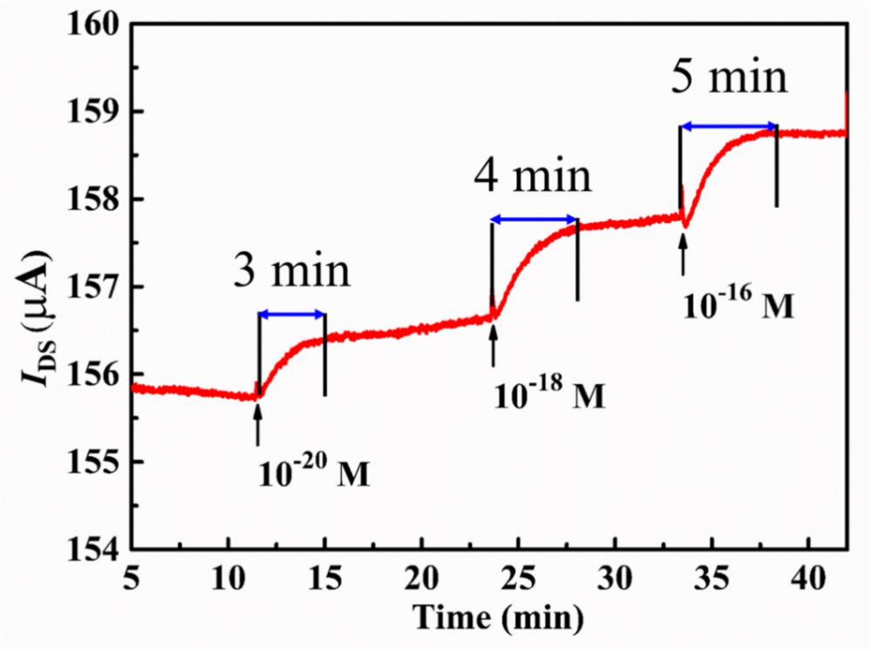

Figure S8 Magnified time scale of channel current response of miRNA-21 concentrations from 10^{-20} M to 10^{-16} M.

Table S1. Comparison of different miRNA detection methods.

Method	Probe	Target	Active Materials	LOD	Linear Rang	Ref.
CV	NH_2-ssDNA	miRNA-141	MWCNTs	8×10^{-15} M	10^{-15}~10^{-8} M	[1]
CV & EIS	Hairpin	miRNA-155	MoC_2	3.3×10^{-13} M	10^{-16}~10^{-9} M	[2]
CV & EIS	PNA	miRNA-21	CHA	2.49×10^{-15} M	10^{-14}~5×10^{-9} M	[3]
DPV	ssDNA	has-miR-486-5p	N-doped LIG	10^{-14} M	10^{-14}~10^{-8} M	[4]
EIS	NH_2-ssDNA	miRNA-21	GCE	3×10^{-15} M	10^{-14}~10^{-8} M	[5]
Fluorescence	FAM-ssDNA	miRNA-21	GO	2×10^{-10} M	2.5×10^{-8}~10^{-7} M	[6]
Fluorescence	polyC-MB	let-7b	MoS_2	3.4×10^{-15} M	10^{-14}~10^{-11} M	[7]
Fluorescence	MB	miRNA-21	GO	3×10^{-11} M	10^{-10}~10^{-8} M	[8]
SPR	ssDNA	miRNA-21	AuNPs	10^{-17} M	10^{-17}~10^{-11} M	[9]
SGGT	Thiol-RNA	miRNA-21	TIPS-pentacene	3×10^{-11} M	10^{-11}~3×10^{-10} M	[10]
SGGT	PNA	let-7b	R-GO	10^{-14} M	10^{-15}~10^{-10} M	[11]
SGGT	ssDNA	miRNA	CVD-grown graphene	10^{-16} M	10^{-16}~10^{-12} M	[12]
SGGT	ssDNA	miRNA-4484	CVD-grown graphene	10^{-14} M	10^{-14}~10^{-12} M	[13]
SGGT	NH_2-ssDNA	miRNA	CVD-grown graphene	10^{-10} M	10^{-10}~10^{-7} M	[14]
SGGT	NH_2-ssDNA	miRNA	GO	10^{-11} M	10^{-11}~10^{-6} M	[15]
SGGT	NH_2-ssDNA	miRNA	CVD-grown graphene	6×10^{-18} M	6×10^{-18}~10^{-6} M	[16]
SGGT	NH_2-ssDNA	miRNA	CVD-grown graphene	2×10^{-12} pM	2×10^{-12}~10^{-6} M	[17]
SGGT	SH-ssDNA	miRNA-21	CVD-grown graphene	10^{-20} M	10^{-20} ~10^{-12} M	This work

EIS: Electrochemical Impedance Spectroscopy, CV: Cyclic Voltammetry, DPV：Differential pulse voltametric, MWCNTs: Multi-walled carbon nanotubes, polyC: poly-cytosine, MB: molecular beacons, SPR: surface plasmon resonance, CHA: Catalytic hairpin assembly, N-doped LIG: N-doped laser induced graphene, GCE: glassy carbon electrode, GO：Graphene oxide.

Table S2. Comparison of medical diagnosis and miRNA-21 detection results from 20 individuals.

Clinical samples	Age	PSA (ng/mL)	Dirac voltage shift (mV)	medical diagnosis	Results based on miRNA-21 detection
1	71	>100	110	Prostate cancer	Prostate cancer
2	63	53.21	100	Prostate cancer	Prostate cancer
3	72	>100	94	Prostate cancer	Prostate cancer
4	74	10.21	90	Prostate cancer	Prostate cancer
5	66	**3.92**	73	Prostate cancer	Prostate cancer
6	52	>100	70	Prostate cancer	Prostate cancer
7	67	**7.187**	70	Prostate cancer	Prostate cancer
8	74	**5.429**	60	Prostate cancer	Prostate cancer
9	74	56.008	60	Prostate cancer	Prostate cancer
10	73	**0.152**	60	Prostate cancer	Prostate cancer
11	69	**22.312**	30	BPH	Normal
12	72	**6.068**	13	Prostate prostatitis	Normal
13	83	**5.442**	12	Prostate prostatitis	Normal
14	75	**3.459**	12	BPH	Normal
15	69	0.341	10	Healthy control	Normal
16	68	**57.36**	10	Healthy control	Normal
17	66	**10.185**	**10**	BPH	Normal
18	64	6.241	10	BPH	Normal
19	71	0.213	8	Healthy control	Normal
20	65	5.007	5	BPH	Normal

Table S3. The ssDNA and miRNA sequences used in this work.

**Names**	**Sequences (5'-3')**
Probe ssDNA strand	Thiol-TCA ACA TCA GTC TGA TAA GCT A
Target miRNA-21	UAG CUU AUC AGA CUG AUG UUG A
miRNA-141	UAA CAC UGU CUG GUA AAG AUG G
One-base mismatched	UAG CUU AUC AGA AUG AUG UUG A
Cy3-Target miRNA-21	Cy3-UAG CUU AUC AGA CUG AUG UUG A

References：

[1] H. V. Tran, B. Piro, S. Reisberg, L. D. Tran, H. T. Duc, M. C. Pham, Label-free and reagentless electrochemical detection of microRNAs using a conducting polymer nanostructured by carbon nanotubes: application to prostate cancer biomarker miR-141, *Biosens. Bioelectron.* **2013**, 49, 164-169.

[2] J. Zhang, X. Hun, Electrochemical determination of miRNA-155 using molybdenum carbide nanosheets and colloidal gold modified electrode coupled with mismatched catalytic hairpin assembly strategy, *Microchem. J.* **2019**, 150, 104095.

[3] P. Fu, S. Xing, M. Xu, Y. Zhao, C. Zhao, Peptide nucleic acid-based electrochemical biosensor for simultaneous detection of multiple microRNAs from cancer cells with catalytic hairpin assembly amplification, *Sens. Actuators, B* **2020**, 305, 127545.

[4] Z. F. Wan, M. Umer, M. Lobino, D. Thiel, N. T. Nguyen, A. Trinchi, M. J. A. Shiddiky, Y. S. Gao, Q. Li, Laser induced self-N-doped porous graphene as an electrochemical biosensor for femtomolar miRNA detection, *Carbon* **2020**, 163, 385-394.

[5] H. Asadi, R. P. Ramasamy, Graphene-based electrochemical biosensor for impedimetric detection of miRNAs as potential cancer biomarkers, *J. Electrochem. Soc.* **2020**, 167, 167523.

[6] M. S. Hizir, M. Balcioglu, M. Rana, N. M. Robertson, M. V. Yigit, Simultaneous detection of circulating oncomiRs from body fluids for prostate cancer staging using nanographene oxide, *ACS Appl. Mater. Interfaces* **2014**, 6, 14772-14778.

[7] M. Xiao, A. R. Chandrasekaran, W. Ji, F. Li, T. Man, C. Zhu, X. Shen, H. Pei, Q. Li,

L. Li, Affinity-modulated molecular beacons on MoS_2 nanosheets for microRNA detection, *ACS Appl. Mater. Interfaces* **2018**, 10, 35794-35800.

[8] L. Yang, B. Liu, M. Wang, J. Li, W. Pan, X. Gao, N. Li, B. Tang, A highly sensitive strategy for fluorescence imaging of microRNA in living cells and in vivo based on graphene oxide-enhanced signal molecules quenching of molecular beacon, *ACS Appl. Mater. Interfaces* **2018**, 10, 6982-6990.

[9] T. Xue, W. Liang, Y. Li, Y. Sun, Y. Xiang, Y. Zhang, Z. Dai, Y. Duo, L. Wu, K. Qi, B. N. Shivananju, L. Zhang, X. Cui, H. Zhang, Q. Bao, Ultrasensitive detection of miRNA with an antimonene-based surface plasmon resonance sensor, *Nat. Commun.* **2019**, 10, 28.

[10] M. Selvaraj, P. Greco, M. Sensi, G. D. Saygin, N. Bellassai, R. D'Agata, G. Spoto, F. Biscarini, Label free detection of miRNA-21 with electrolyte gated organic field effect transistors (EGOFETs), *Biosens. Bioelectron.* **2021**, 182, 113144.

[11] B. Cai, L. Huang, H. Zhang, Z. Sun, Z. Zhang, G. Zhang, Gold nanoparticles-decorated graphene field-effect transistor biosensor for femtomolar microRNA detection, *Biosens. Bioelectron.* **2015**, 74, 329-334.

[12] M. Tian, S. Xu, J. Zhang, X. Wang, Z. Li, H. Liu, R. Song, Z. Yu, J. Wang, RNA detection based on graphene field-effect transistor biosensor, *Adv. in Condensed Mat. Phy.* **2018**, 2018, 1-6.

[13] J. Gao, Y. Gao, Y. Han, J. Pang, C. Wang, Y. Wang, H. Liu, Y. Zhang, L. Han, Ultrasensitive label-free miRNA sensing based on a flexible graphene field-effect transistor without functionalization, *ACS Appl. Electron. Mater.* **2020**, 2, 1090-1098.

[14] R. Song, M. Tian, Y. Li, J. Liu, G. Liu, S. Xu, J. Wang, Detection of microRNA based on three-dimensional graphene field-effect transistor biosensor, *Nano* **2020**, 15, 2050039.

[15] M. Sun, C. Zhang, J. Wang, C. Sun, Y. Ji, S. Cheng, H. Liu, Construction of high stable All-graphene-based FETs as highly sensitive dual-signal miRNA sensors by a covalent layer-by-layer assembling method, *Adv. Electron. Mater.* **2020**, 6.

[16] M. T. Hwang, M. Heiranian, Y. Kim, S. Y. You, J. Y. Leem, A. Taqieddin, V. Faramarzi, Y. H. Jing, I. Park, A. M. van der Zande, S. Nam, N. R. Aluru, R. Bashir, Ultrasensitive detection of nucleic acids using deformed graphene channel field effect biosensors, *Nat. Commun.* **2020**, 11, 1543.

[17] M. Tian, M. Qiao, C. Shen, F. Meng, L. A. Frank, V. V. Krasitskaya, T. Wang, X. Zhang, R. Song, Y. Li, J. Liu, S. Xu, J. Wang, Highly-sensitive graphene field effect transistor biosensor using PNA and DNA probes for RNA detection, *Appl. Surf. Sci.* **2020**, 527.
